# A Comparison of the Ability of Levels of Urinary Biomarker Proteins and Exosomal mRNA to Predict Outcomes after Renal Transplantation

**DOI:** 10.1371/journal.pone.0098644

**Published:** 2014-06-11

**Authors:** Philip W. Peake, Timothy J. Pianta, Lena Succar, Mangalee Fernando, Debbie J. Pugh, Kathleen McNamara, Zoltan H. Endre

**Affiliations:** 1 Department of Nephrology, Prince of Wales Hospital, Sydney, Australia; 2 Prince of Wales Clinical School, University of New South Wales, Sydney, Australia; The University of Manchester, United Kingdom

## Abstract

**Background:**

mRNA for biomarkers of kidney injury extracted from urinary exosomes may reflect or predict levels of the corresponding protein after transplantation and clinical outcomes.

**Methods:**

Urinary exosomes were isolated from patients following renal transplantation, from healthy controls, and patients with CKD. Expression of exosomal mRNA for the injury biomarkers neutrophil gelatinase-associated lipocalin (NGAL), interleukin-18 (IL-18), kidney injury molecule-1 (KIM-1), and cystatin C was compared with the concentrations of corresponding urinary proteins, 18S RNA and serum creatinine.

**Results:**

All biomarker protein concentrations increased after transplantation, and urinary NGAL and IL-18 at 24 and 168 h correlated with the day 7 creatinine reduction ratio (CRR). Exosomal18S RNA increased after transplantation, but exosomal mRNA for NGAL, IL-18 and cystatin C did not correlate with the day 7 CRR, or urinary biomarker concentrations at any time after transplantation. Exosomal NGAL mRNA was lower 4 h after transplantation than in control exosomes. In contrast, exosomal mRNA for cystatin C was unchanged after transplantation and in CKD, although urinary cystatin C temporarily increased following transplantation. Urinary KIM-1 increased after transplantation, but exosomal mRNA for KIM-1 remained undetectable. In CKD 18S RNA was raised, and exosomal mRNA for NGAL, IL-18 and cystatin C was detected in all patients. While urinary NGAL was greater in CKD than control subjects, exosomal NGAL mRNA was unchanged. Exosomal IL-18 mRNA was increased in CKD, but not IL-18 protein.

**Conclusions:**

After renal transplantation, urinary NGAL and IL-18 levels reflect the day 7 CRR. However, while mRNA for these biomarkers is present in exosomes, their levels do not reflect or predict urinary biomarker levels or the CRR. This likely reflects the fact that packaging of mRNA in exosomes is selective, and is not necessarily representative of mRNA in the parent cells responsible for biomarker production.

## Introduction

Renal transplantation is inevitably associated with a period of renal ischemia and probable exposure to nephrotoxins, and the resulting kidney injury can manifest as delayed or slow graft function. However, creatinine has a long half life and a large volume of distribution, factors which prevent it from providing both a real-time and accurate measure of kidney function and injury [1]. While alternative serum biomarkers have been proposed, including plasma cystatin C for renal function and neutrophil gelatinase-associated lipocalin (NGAL) [2], [3], urinary biomarkers may provide more specific evidence of tubular injury, including cystatin C, NGAL, Kidney Injury Molecule-1 (KIM-1) and interleukin 18 (IL-18). Previous studies show that urinary injury biomarkers, especially IL-18 and NGAL correlate with graft outcome, but only at 24 hours when intervention is unlikely to be helpful or when serum creatinine [sCr] is also predictive [4]. Thus the search for new biomarkers of kidney injury remains ongoing [1], [5]–[7].

Urinary exosomes, small membrane-bound vesicles of 50–130 nm diameter shed by all tubular cells are a potential new source of biomarkers. They contain cytoplasmic mRNA, which is not normally detectable in the RNAase-containing urine [8]–[10], but remains stable within the exosomal lipid bilayer [9], [11]–[14]. We hypothesised that exosomal mRNA for biomarkers of renal injury would reflect or predict the concentrations of the corresponding urinary protein, and/or predict graft outcome [15]–[17]. We examined levels of exosomal mRNA for the inducible proteins NGAL, a renoprotective protein known to bind siderophores, KIM-1, a transmembrane protein which imparts a phagocytic phenotype to proximal tubule cells, and the inflammatory marker IL-18, and compared these levels with those of the corresponding urinary protein. These findings were compared with those for the constitutively produced cystatin C, whose presence in urine reflects impaired low molecular weight protein uptake by the proximal tubule [18]. Levels of urinary protein and exosomal mRNA for these biomarkers were determined in normal subjects and compared with those in patients immediately following renal transplantation, and with the day 7 creatinine reduction ration [CRR] as a measure of clinical outcomes. As a positive control, levels of urinary protein and exosomal mRNA for these biomarkers were compared with the corresponding levels in those with chronic kidney disease [CKD] complicated by significant proteinuria. Levels of NGAL and IL-18 protein, but not exosomal mRNA correlated with the day 7 CRR, and mRNA expression did not predict urinary biomarker concentrations in normal or pathological situations. However, mRNA for IL-18 did detect ongoing pathology in CKD not reflected in levels of the urinary protein. These data suggest levels of exosomal mRNA for three commonly used protein biomarkers of kidney injury were not related to urinary levels of the corresponding proteins, or to clinical outcomes.

## Subjects and Methods

### Patients

We prospectively consented consecutive patients of the renal transplantation service at the Prince of Wales Hospital, Sydney and normal volunteers. The study was approved by the South Eastern Sydney Local Health District Human Research Ethics Committee [HREC 10/113]. All participants gave written informed consent to participate in the study. Urine samples were obtained from groups of non-oliguric adults aged 18 or older (*Table 1*).

**Table 1:** Clinical characteristics of study subjects.

	Normal (11)	Acute Transplant (14)	CKD (9)
**Female**	7 (63)	5 (36)	3 (30)
**Age**	34 (28–45)	46 (32–58)	61 (54–76)
**S Creatinine (µmol/L)**	62 (54–79)	NA	313 (208–468)
**Day 7 CRR**	NA	−0.74 (−0.83–−0.52)	NA
**eGFR (mL/min/1.73 m^2^)**	>60 (all subjects)	NA	13 (12–31)
**uACR (mg/mmol Cr)**	0.79 (0.47–6.6)	NA	620 (203–1495)
**Etiology of CKD**			
Glomerulonephritis	NA	7 (50)	2 (20)
Diabetes	NA	2 (14)	6 (60)
Polycystic kidney disease	NA	2 (14)	0
Ischaemia	NA	1 (7)	0
Other	NA	2 (14)	2 (20)
**Medications: Antiproliferative**			
Mycophenolate	0	14	0
Azathioprine	0	0	0
Nil	11	0	10
**Medications: Calcineurin inhibitor**			
Cyclosporine	0	7	0
Tacrolimus	0	7	0
Nil	11	0	10
**Medications: Sirolimus**	0	0	0

Key: Data are median (IQR), or n (%).

NA: not applicable, eGFR: estimated glomerular filtration rate (Modification of Diet in Renal Disease formula), uACR: albumin: creatinine ratio.

The Acute Transplant group were recipients of a renal transplant (8 deceased donors, 6 live donors). Before transplant, all these patients were dialysis dependent, and 8/14 were anuric; the remainder had some residual urine output. Induction of immunosuppression consisted of methylprednisolone, mycophenolate sodium and basiliximab in all patients and either cyclosporine (n = 7) or tacrolimus (n = 7) at the treating physician’s discretion. The CRR was defined by the difference between the sCr collected within 1 h after surgery (baseline) and the sCr at day 7 divided by baseline sCr.

A normal (volunteer) group undertook a screening questionnaire and investigations to exclude any CKD. Exclusion criteria comprised a history of hypertension, diabetes, smoking or CKD, the regular use of medications including non-steroidal anti-inflammatory drugs; resting blood pressure ≥140/90 mmHg; abnormal urinalysis; or estimated glomerular filtration rate (by Modification of Diet in Renal Disease (*MDRD*) formula) <60 mL/min/1.73 m^2^ (CKD).

A comparator chronic kidney disease group comprised general nephrology patients with CKD (eGFR <60 ml/min/1.73 m^2^) and proteinuria (>1g/days, see Table 1).

### Sample Preparation and Isolation Methods

In brief, urine for exosome analysis was centrifuged at 3,200 g for 10 min to remove cellular debris and then stored at −80°C until analysis [9], [19]. In the Acute Transplantation group, samples were taken at 4, 24 and 168 h post-operatively. Separate urine samples were collected into protease inhibitor solution [Complete protease inhibitor, Roche, Sydney Australia].

### Exosome Purification and Analysis

Urine samples were vortexed, then centrifuged at 3200 g for 15 min. The supernatant (25 ml) was centrifuged at 3200 g in Vivaspin 20 nanomembrane concentrators (MWCO 100 kDa; Sartorius, Goettingen, Germany) to collect the exosomal fraction [19]. RNA in the retentate was twice purified by Trisure following the manufacturer’s protocol [Bioline, Sydney, Australia]. cDNA was generated using the Tetro cDNA Synthesis kit [Bioline, Sydney, Australia] following the manufacturer’s protocols using random primers. RNA was quantitated with commercially designed Taqman probes and reagents [Applied Biosystems, Melbourne Australia] by PCR performed on a Corbett 3000 real-time machine. All mRNA probes were exon spanning to exclude the possibility of DNA contamination in the preparations, and were Hs00183813_m1 for ALIX, Hs01008571_m1 for NGAL, Hs01038788_m1for IL-18, Hs99999901_s1 for 18S RNA, Hs00273334_m1 for KIM-1, and Hs00264679_m1 for cystatin C.

Quantitation was based upon a dilution series of cDNA from 18S RNA. Results for levels of cDNA for cystatin C, NGAL, KIM-1 and IL-18 were calculated relative to the levels of 18S for each cDNA preparation using the comparative Ct method [20]. Each preparation of RNA from urine was dissolved in 25 µl water, providing sufficient RNA for cDNA synthesis for 5 gene assays in duplicate. Data for mRNA levels in transplant patients were compared with levels of mRNA in normal subjects, and with those in patients with CKD as a positive control.

Assays were accepted for analysis where duplicate values were concordant, the 1/100 dil. of 18S RNA gave Ct <31 and mRNA results for biomarker cDNA gave Ct <37. The lowest detectable concentration based on our data was calculated as 0.000061 for 1/2^Ct^. cDNA amplification of mRNA showed efficiencies of 100±10%, and between assay CV% was <2%. An experiment utilising HK-2 proximal tubular cells in culture served as a positive control for mRNA detection.

### Biomarker Assays

Biomarkers in urine were measured quantitatively by sandwich ELISA according to the manufacturer’s instructions using commercial ELISA kits (R&D Systems). The urine samples were mixed on collection with Complete protease inhibitor [Roche] in a ratio of 2∶1, and stored at 4°C until collection. On receipt samples were centrifuged, aliquoted to avoid repeated freeze thaw cycles and stored at −80°C. All measurements were done in duplicate, with ELISA plates read at 450 nm. Data collected was analyzed using Prism 6 [GraphPad] curve-fitting equations. The lowest detectable concentration of each antigen was designated to be half the concentration of the lowest standard. Creatinine levels were determined by commercial enzymatic reagents and standards [ThermoFisher, Melbourne Australia].

### Statistical Analysis

Data were in general not normally distributed, and non-parametric one way Kruskal-Wallis ANOVA and Dunn’s post test analyses were performed with significance adjusted for multiple comparisons. Nonparametric Spearman coefficients were calculated to determine relationships between parameters. Prism [Version 6, GraphPad Software, La Jolla, CA] was used for all statistical analyses. P<0.05 was considered significant using two-tailed analyses.

## Results

### Subjects

The clinical demographics of the groups: normal volunteers, acute transplant, and patients with chronic kidney disease are summarised in Table 1. No transplant patients showed evidence of acute rejection.

### Urinary Biomarker Protein Concentrations Increased after Transplantation

**Figure 1:**
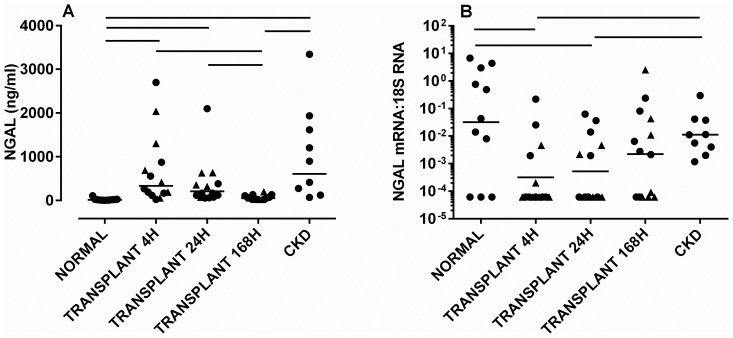
Concentration of [A] NGAL in whole urine, and [B] concentration of mRNA for NGAL in exosomes. Samples were taken at 4, 24 and 168[cf.deceased] donors are shown as triangles. Significant differences between groups [p<0.05] are denoted by horizontal lines.

Increased urinary concentrations of NGAL and IL-18 at D1 and D7, (but not at 4 h), correlated with the day 7 CRR [Table 2]. Cystatin C and KIM-1 also increased after transplantation but did not correlate with early graft outcome. Urinary NGAL protein rose after transplantation, was higher at 4 and 24 h than in normal volunteers [both p<0.001]. Levels had returned to normal by 168 h [Fig. 1A]. NGAL protein was higher in both CKD patients [p<0.001] and at 168 h after transplantation [p<0.001] than in control subjects.

**Table 2:** Spearman correlations between levels of protein and mRNA biomarkers and the day 7 creatinine reduction ratio at 4, 24 and 168

	4 h post transplantation		24 h post transplantation		168 h post transplantation	
	r_s_	p	r_s_	p	r_s_	p
**Protein**						
NGAL	−0.36	0.20	−0.62	0.02	−0.60	0.02
IL-18	−0.31	0.14	−0.48	0.03	−0.70	<0.0001
CysC	−0.43	0.12	−0.17	0.54	−0.42	0.10
KIM-1	0.17	0.57	0.15	0.63	0.26	0.39
**mRNA**						
NGAL	0.15	0.61	0.33	0.24	−0.08	0.25
IL-18	0.04	0.52	0.18	0.25	0.09	0.62
CysC	0.39	0.17	0.21	0.47	0.03	0.91

There were no significant correlations between the day 7 CRR and mRNA for these biomarkers, or between mRNA and protein levels of biomarkers. KIM-1 mRNA was not reliably detected in exosomes.

**Figure 2:**
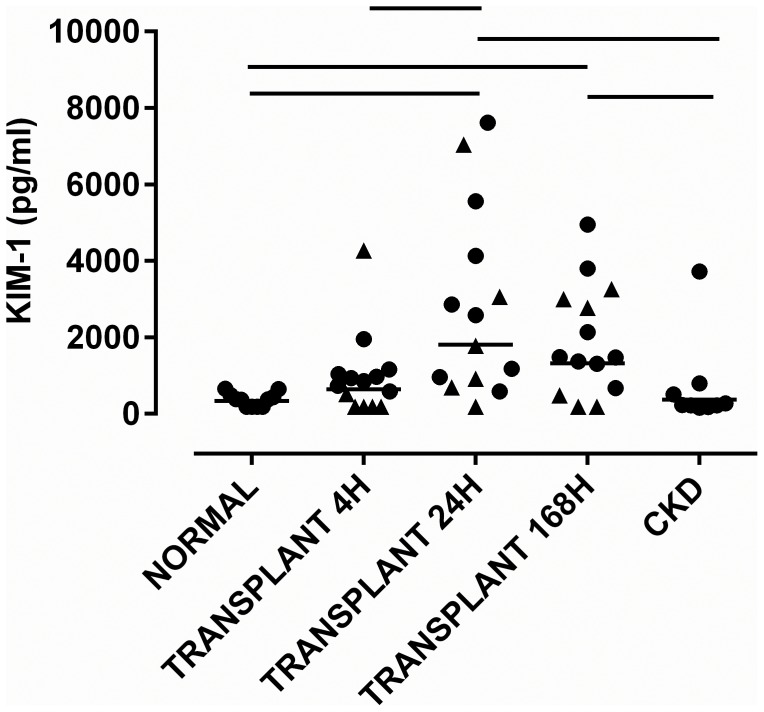
Concentration of [A] KIM-1 in whole urine 4, 24 and 168 h after renal transplantation. Samples were taken at 4, 24 and 168-1 was not detected in most subjects. For the transplant patients, the data for those who received kidneys from live donors [cf. deceased] are shown as triangles. The geometric mean is shown. Significant differences between groups [p<0.05] are denoted by horizontal lines.

Urinary KIM-1 increased after transplantation peaking at 24 h and remained higher than in control subjects168 h after transplantation [p<0.001 and p<0.01 respectively] [Fig. 2 ]. Levels 24 and 168 h after transplantation were higher than in CKD patients with proteinuria [p<0.01 and p<0.05 respectively].

**Figure 3:**
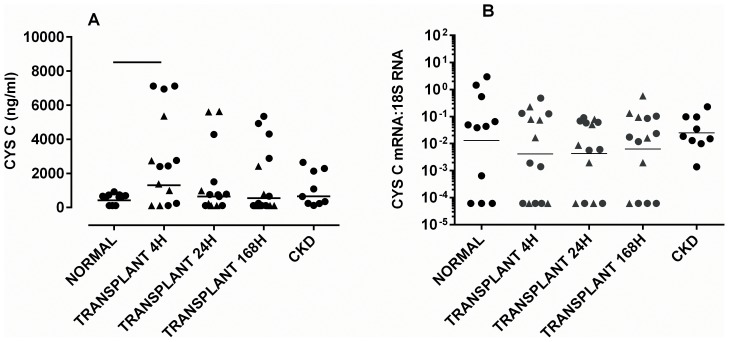
Concentration of [A] cystatin C in whole urine, and [B] concentration of mRNA for cystatin C in exosomes. Samples were taken at 4, 24 and 168[cf. deceased] are shown as triangles. The geometric mean is shown. Significant differences between groups [p<0.05] are denoted by horizontal lines.

Levels of urinary cystatin C protein were higher 4 h after transplantation than in control subjects, [p<0.05] but were not increased at other points in time, or in CKD patients with proteinuria [Fig. 3A].

**Figure 4:**
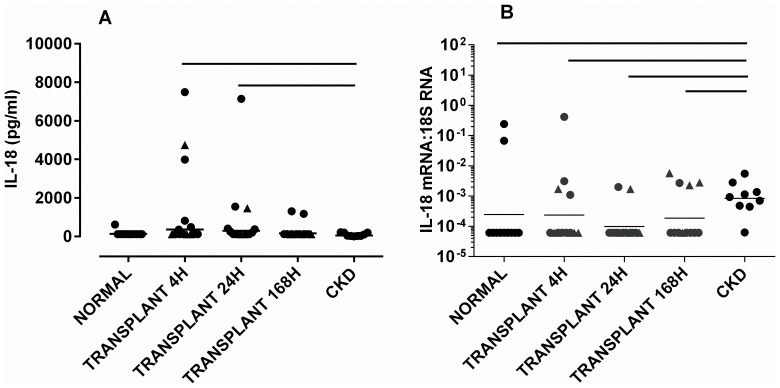
Concentration of [A] IL-18 in whole urine, and [B] concentration of mRNA for IL-18 in exosomes. Samples were taken at 4, 24 and 168[cf. deceased] donors are shown as triangles. The geometric mean is shown. Significant differences between groups [p<0.05] are denoted by horizontal lines.

The median urinary IL-18 protein concentration increased after transplantation, and was higher 4 and 24 h after transplantation compared to CKD patients [both p<0.05] [Fig. 4A].

### Exosome Production Increased after Transplantation and in CKD with Proteinuria

**Figure 5:**
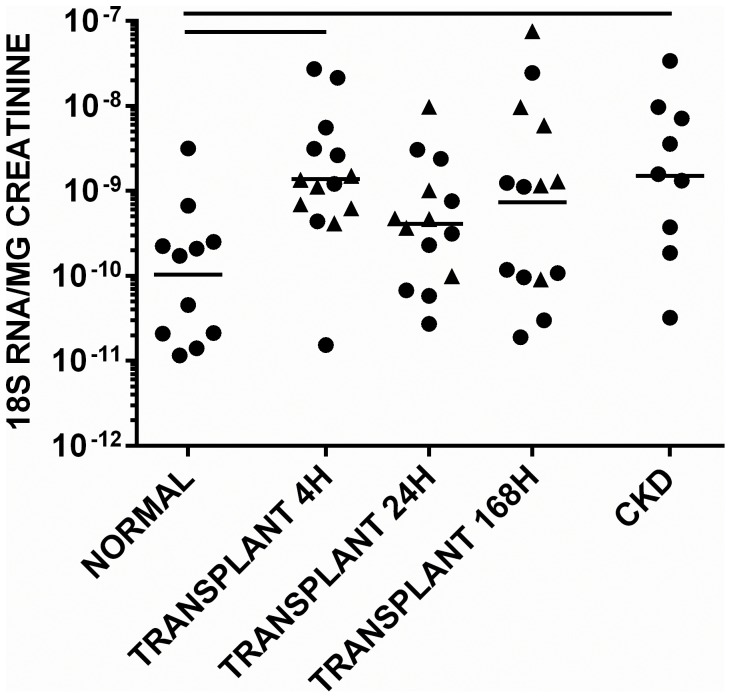
Concentration of 18S RNA in urinary exosomes. Samples were taken at 4, 24 and 168[cf. deceased] are shown as triangles. The geometric mean is shown. Significant differences between groups [p<0.05] are denoted by horizontal lines.

Exosomal 18S RNA was present in urine from all subjects. Levels in acute transplant patients at 4 h and in CKD patients were greater than in control subjects, [p<0.05 for both] suggesting increased exosome production [Fig. 5]. However, levels did not correlate with the day 7 CRR. 18S RNA was generally present 100 fold or more times the concentration of mRNA for each biomarker. Exosomal mRNA for the inducible proteins NGAL, IL-18 and cystatin C was detected in all subjects with CKD. In each population, mRNA levels were normalised to those of 18S RNA, with results similar to those seen when raw Ct PCR values from RNA preparations were compared. In each case RNA preparations were derived from 25 ml urine. To confirm the identity of the source of the RNA preparations, mRNA for the exosome biomarker ALIX [ =  programmed cell death 6–interacting protein] was demonstrated to be present [not shown].

### NGAL Exosomal mRNA Decreased after Transplantation

Exosomal mRNA for NGAL was decreased at 4 and 24 h post transplantation compared with controls, but had increased to control levels by 168 h [p<0.05] [Fig. 1B]. All CKD subjects had exosomal mRNA for NGAL, and this was present at levels equivalent to controls. There was no correlation between levels of mRNA for NGAL and CRR, or with the corresponding levels of protein 4 h after transplantation or at later times, regardless of whether absolute levels of mRNA or levels adjusted for 18S RNA were used.

### Exosomal mRNA for KIM-1 was Undetectable after Transplantation

mRNA for KIM-1 was not detected in normal volunteers and was detected [at low levels] in only three subjects in other groups. Assay performance was verified by ready detection of mRNA for KIM-1 in positive control HK-2 proximal tubular cells (data not shown).

### Cystatin C Exosomal mRNA Remained Unchanged after Transplantation

There were no significant differences between exosomal mRNA levels for cystatin C in all groups [Fig. 3B], and no significant correlation between levels of mRNA and CRR or with cystatin C protein.

### IL-18 Exosomal mRNA Remain Unchanged after Transplantation

Exosomal IL-18 mRNA was significantly higher in CKD patients than in normal volunteers [p<0.05], and higher than in transplant recipients at 4 h [p<0.05], 24 h [p<0.01] and 168 h [p<0.05] [Fig. 4B]. There were no significant differences between mean levels in transplant patients at different times. There was no significant correlation between levels of mRNA for IL-18 and the D7 CRR, or the corresponding levels of protein.

## Discussion

This study demonstrates for the first time that mRNAs for biomarkers of renal cellular injury including NGAL, cystatin C and IL-18 were present in urinary exosomes from normal subjects, patients at various times after renal transplantation, and in subjects with CKD. After renal transplantation, free urinary protein concentrations of NGAL and IL-18 correlated with the day 7 CRR. However, there was a poor correlation between exosomal mRNA for the same injury biomarkers and the day 7 CRR, and with biomarker protein levels.

18S RNA, a ‘house-keeping’ gene product, was detected in all preparations of urinary exosomes, at levels 100 fold or more higher than biomarker mRNA concentrations. 18S RNA concentrations were higher after acute transplantation and in CKD patients with proteinuria than in normal volunteers, a finding consistent with an increased production of exosomes. Such data parallel the increased concentrations of serum exosomes observed in patients with cancer, hypoxia or inflammatory disease compared with those in healthy individuals [9], [21], [22], and with the recently observed increase in 18S RNA in urinary cells collected prior to and during episodes of renal transplant rejection [23].

As expected, the urinary protein concentrations of KIM-1, NGAL, cystatin C and IL-18 increased soon after kidney transplantation and then declined. The KIM-1 peak was delayed (24 h) compared with NGAL and cystatin C, both of which peaked 4 h after clamp release. Levels of NGAL and IL-18 correlated with the day 7 CRR at 24 and 168 h post transplantation, a finding reported in other kidney transplantation studies [4]. By contrast, exosomal mRNA for NGAL fell immediately after renal transplantation, then rose to normal levels after 168 h, but levels did not correlate with the day 7 CRR. In contrast, exosomal mRNA for NGAL was high in normal volunteers, especially given the low concentration of NGAL protein in the same urine.

NGAL mRNA is largely synthesized in the distal nephron [24], from where most urinary NGAL protein derives [25], and rises in acute injury [26]. While NGAL protein is freely filtered by the glomerulus, it is normally reabsorbed by the proximal tubule, but urine levels may be affected by competition with other proteins for megalin-cubulin uptake in the proximal tubule [18], [27].

Our finding that levels of exosomal NGAL mRNA do not reflect urinary NGAL is consistent with reports that constituents in exosomes from the same cell may vary over time [28], and represent only a subset of the constituents of the parent cell [9], [11]. For example, urinary exosomes contain many apical but few basolateral membrane proteins, and exosomes from Jurkat cells in which mRNA for IL-2 is upregulated have an absence of detectable mRNA for IL-2 in their exosomes [29]. Such selectivity may reflect a role for exosomes in cell-cell communication [12], [30]: studies have established physiological effects on target cells as a result of the incorporation of exosomal content, including both protein and RNA [9], [22].

The disconnection between urinary protein and exosomal mRNA was also observed in KIM-1 and IL-18. Exosomal mRNA for KIM-1 remained undetectable in most subjects although KIM-1 mRNA has been detected in cells from the urinary sediment of patients with kidney graft dysfunction [31]. However, this source did not include exosomes and has a different mRNA profile to that found in exosomes [32]. Similarly, while levels of mRNA for IL-18 rose after transplantation, they did not correlate with levels of the corresponding protein, or with the day 7 CRR. The latter study also demonstrated mRNA was internal to exosomes, given the presence of RNAases in the urine, and the resistance of exosomal RNA to the experimental addition of RNAases during isolation of exosomes.

The data for these inducible proteins, which largely derive from renal synthesis, may be contrasted with the constitutively produced cystatin C, which is produced at a constant rate by all nucleated cells [33]. Serum cystatin C is freely filtered at the glomerulus and usually completely reasborbed in the proximal tubule. Increases in urinary cystatin C protein observed after transplantation therefore reflect proximal tubular injury [34] as well as competition by other proteins for megalin-cubulin uptake [18], [27]. In this study, urinary cystatin C protein had increased by 4 h post transplantation. However, exosomal cystatin C mRNA levels remained stable after transplantation. The relatively constant amounts of exosomal mRNA for cystatin C may reflect differences in the way constitutively produced ‘housekeeping’ mRNA is packaged into exosomes compared with mRNA for inducible biomarkers of kidney injury.

In CKD patients with proteinuria, urinary levels of both NGAL protein and exosomal mRNA for NGAL were increased. These subjects expressed the highest observed levels of NGAL protein. However, NGAL protein concentrations did not correlate with exosomal mRNA levels. In comparison, levels of IL-18 were low, while exosomal mRNA for IL-18 was detectable in all patients with CKD and at higher levels than acutely after transplantation. However, exosomal mRNA for IL-18 was detectable in only two of 11 healthy control subjects. The raised levels of mRNA for IL-18 in the proteinuric subjects therefore suggest that in CKD raised exosomal IL-18 mRNA reflected ongoing [chronic] renal injury, while urinary IL-18 protein itself did not. The fact that exosomal mRNA for NGAL, IL-18 and cystatin C was detected in all subjects with CKD served as a positive control, and suggested that a failure to detect exosomal mRNA implies very low exosomal levels of biomarker mRNA in these subjects.

In summary, a range of physiological processes independently influences levels of urinary protein and exosomal mRNA, but only urinary biomarker protein correlated with clinical outcomes, such as the day 7 CRR. The data suggest that the incorporation of mRNA for biomarkers into urinary exosomes after renal transplantation does not reflect production of protein biomarkers by renal cells lining the lumen.
